# Configuration of elastin fibers in the intra- and extra-capsule ligaments of the elderly cricoarytenoid joint

**DOI:** 10.1007/s00405-023-08003-y

**Published:** 2023-05-19

**Authors:** Qin Wang, Sheng Li, Huaqiao Wang, Ming Zhang

**Affiliations:** 1 Department of Otolaryngology, The First Affiliated Hospital, Anhui Medical University, Hefei, China; 2 Department of Anatomy, The Zhongshan School of Medicine, Sun Yat-Sen University, Guangzhou, China; 3 Present Address: The Second Affiliated Hospital of Guangzhou University of Chinese Medicine, Guangzhou, 510120 China; 4 Department of Anatomy, University of Otago, Dunedin, New Zealand

**Keywords:** Fibrous configuration, Cricoarytenoid joint, Cricoarytenoid ligaments, Elastic fibers, Elderly cadaver, Verhoeff van Gieson staining

## Abstract

**Purpose:**

To define the localization and configuration of the elastic fibers of the cricoarytenoid ligament (CAL) and their relationship with the cricoarytenoid joint (CAJ) capsule.

**Methods:**

Twenty-four CAJs from twelve cadavers were analyzed using Verhoeff-Van Gieson staining, and immunohistochemistry methods. This is a prospective study.

**Results:**

The CAL was classified into two parts: an extra-capsular anterior-CAL and an intra-capsular posterior-CAL. The both parts contained rich elastic fibers. The elastic fibers of the anterior-CAL were orientated in both anterior–posterior and superior–inferior directions and under a relaxation status, whereas the elastic fibers of the posterior-CAL were arranged in a lateral–medial direction and under a taut status.

**Conclusions:**

This study defined the fine configuration of the CAL, particularly its elastic fibers, which may help us to better understand the biomechanics of the CAJ motions, and differential diagnosis of CAJ disorders. The results of the study re-confirm that the P-CAL is the key posterior–lateral passive force to limit the mobility of the muscular process of the arytenoid cartilage and stabilize the CAJ, whereas the A-CAL may protect the CAJ from an over superior–lateral–posterior motion.

**Level of evidence:** H/A.

## Introduction

The cricoarytenoid joint (CAJ) plays a pivotal role in respiration and phonation. The CAJ permits motion in a gliding, rocking, and rotation fashion [1–5]. In addition, the CAJ also permits a motion of vertical jumping [6, 7]. The movement and stability of the CAJ are provided by both active and passive elements. The active elements, such as the intrinsic and extrinsic laryngeal muscles and their innervations, have been well-studied [5, 8]. The passive elements include the laryngeal cartilages, CAJ capsule and laryngeal ligaments and contribute to the stability and positioning of the CAJ. Of the main passive elements of the CAJ, the cricoarytenoid ligament (CAL) strengthens the CAJ capsule and stabilizes the CAJ. The CAL connects the arytenoid caretilage to the cricoid cartilage and is traditionally termed the posterior cricoarytenoid ligament and considered as a capsule ligament [9]. Unlike most ligaments in the body, the CAL contains rich elastic fibers [6]. Elastic fibers allow a tissue to stretch under load and recoil to its original configuration after load is released [10]. There are very few studies on the configuration of the elastic fibers of the CAJ. In this study, thus, we aimed to define the localization and configuration of the elastic fibers of the CAL and their relationship with the CAJ.

## Materials and methods

Twenty-four CAJ specimens were collected from twelve embalmed cadavers (4 females and 8 males, age range 46–89 years). The cadavers were donated to the department of anatomy for the purposes of teaching and research under the Human Tissues Act, and the approval for the use of the cadavers was granted by the University of Otago Human Ethics Committee (Health) and by the Medical Ethics Committee of Anhui Medical University. The collected specimens were decalcified as recommended by Ted Pella, Inc. (Redding, CA, USA). Briefly, a Pelco BioWave Pro laboratory microwave oven equipped with a thermistor copper temperature probe and a microwave load cooler (Ted Pella Inc, Redding, CA) were used for the decalcification process. The specimen was incubated in an ethylenediaminetetraacetic acid (EDTA) solution (0.27 M EDTA, pH 7.4). Microwave irradiation was set at 150 W, and the temperature was programmed to the maximum of 35 °C. The EDTA solution was replaced every 4 h. The decalcification was completed in 5 days. The decalcified specimen was dehydrated through a series of alcohol and xylene solutions, and then embedded in paraffin. Serial transverse and coronal sections were cut at 7 μm and stained with Verhoeff van Gieson (VG) method, and observed under a light microscope (Olympus AX70 Provis, Tokyo, Japan).

**Fig. 1:**
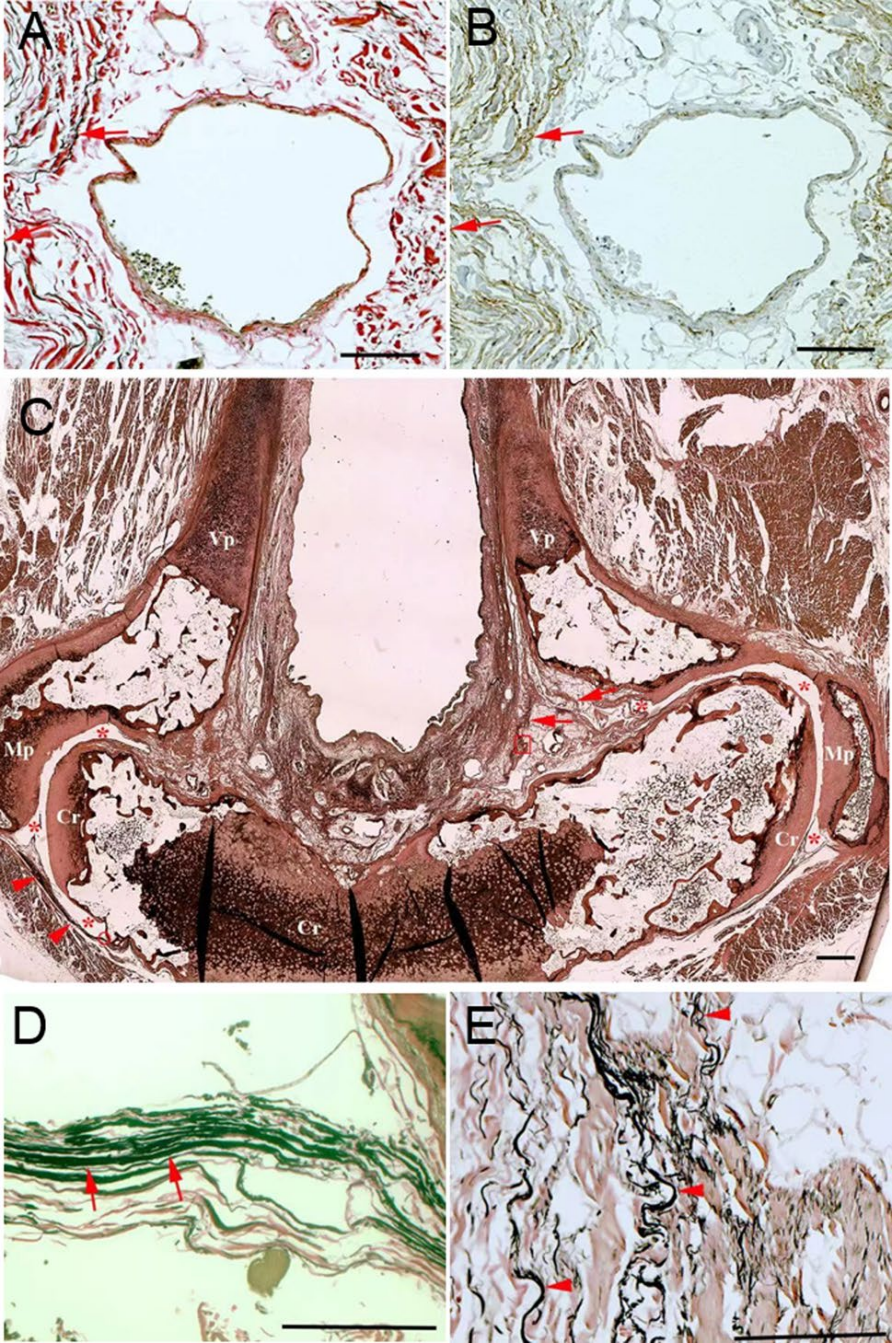
**A**, **B** Verification of elastic fiber staining (arrows) of Verhoeff van Gieson method (**A**) with anti-elasin immunohistochemistry method (**B**). **C** Transverse section at the middle level of the cricoarytenoid joint stained with Verhoeff van Gieson method. **D** and **E** are the high magnification views of the small red circle and box in **C**, showing elastic fibers in the posterior (arrows) and anterior (arrowheads) cricoarytenoid ligaments. *Asterisks* the joint cavity; *Vp* vocal process of the arytenoid cartilage; *Mp* muscular process of the arytenoid cartilage; *Cr* cricoid cartilage; Bars = 1 mm

To verify the specificity of elastic fibers staining with the VG method, the elastin was labeled with the immunohistochemistry method as previously described [11]. In brief, the sections were dewaxed and rehydrated through a series of xylene and alcohol solutions. After washed in PBS, the sections were incubated overnight at 4 °C with a monoclonal anti-elastin antibody (Abcam, Cambridge, UK). The immunoreactivity was then developed using biotinylated-anti-mouse IgG, streptavidin biotinylated-horseradish peroxidase complex and diaminobenzidine as the chromogen. Nonspecific immunohistochemical staining was controlled for by replacing the monoclonal antibody with non-immunized mouse IgG. The sections were examined using an Olympus AX70 microscope. Figure 1A, B demonstrates that the black violet colored structures stained with Verhoeff-Van Gieson method were verified as the specific staining of elastin fibers by the immunohistochemistry method.

## Results

The CAL extended between the base of the arytenoid cartilage and the anterior (A-CAL) or posterior (P-CAL) aspects of the cricoid lamina (Fig. 1C). Both A-CAL and P-CAL contained rich elastin fibers but their locations, orientations and relationship with the CAJ were different.

### The elastic fibers anterior and medial to the CAJ

**Fig. 2:**
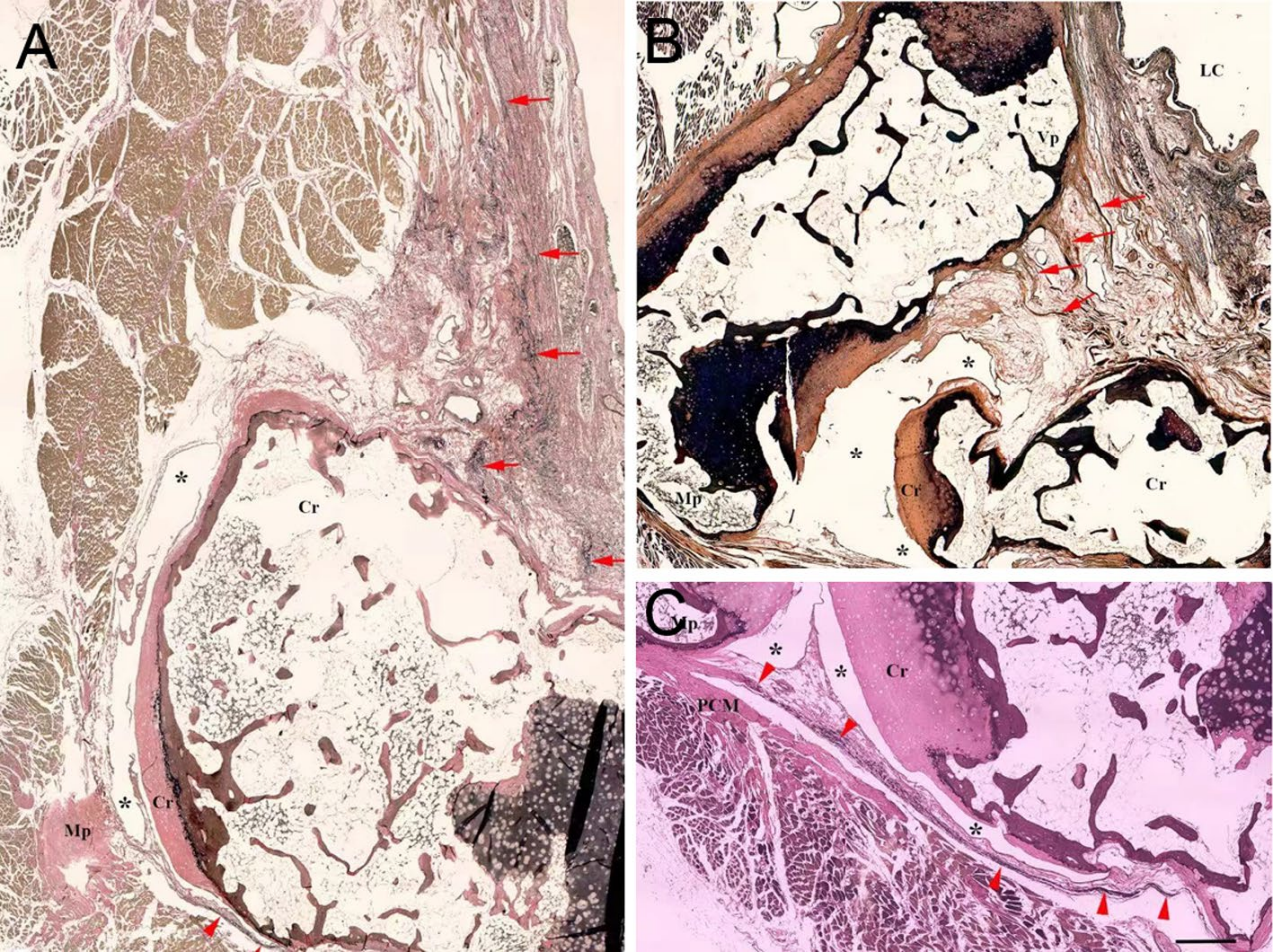
Three transverse sections at the lower (**A**), upper (**B**) and middle (**C**) levels of the cricoarytenoid joint, stained with Verhoeff van Gieson method. **A** At the level of the lower end of the muscular process (Mp) of the arytenoid cartilage, the elastic fibers are distributed anterior–medially (arrows) and posteriorly (arrowheads) to the joint cavity (asterisks). **B** At the upper end of the joint (asterisks), the anterior cricoarytenoid ligament (arrows) extends between the base of the vocal process (Vp) of the arytenoid cartilage and the anterior surface of the cricoid cartilage (Cr). *LC* laryngeal cavity. **C** At the middle level of the joint, the elastic fibers (arrowheads) are dominant in the posterior cricoarytenoid ligament which shares a common attachment with the posterior cricoarytenoid muscle (PCM) on the muscular process of the arytenoid cartilage. *Asterisks* joint cavity; *Cr* cricoid cartilage; Bars = 1 mm

**Fig. 3:**
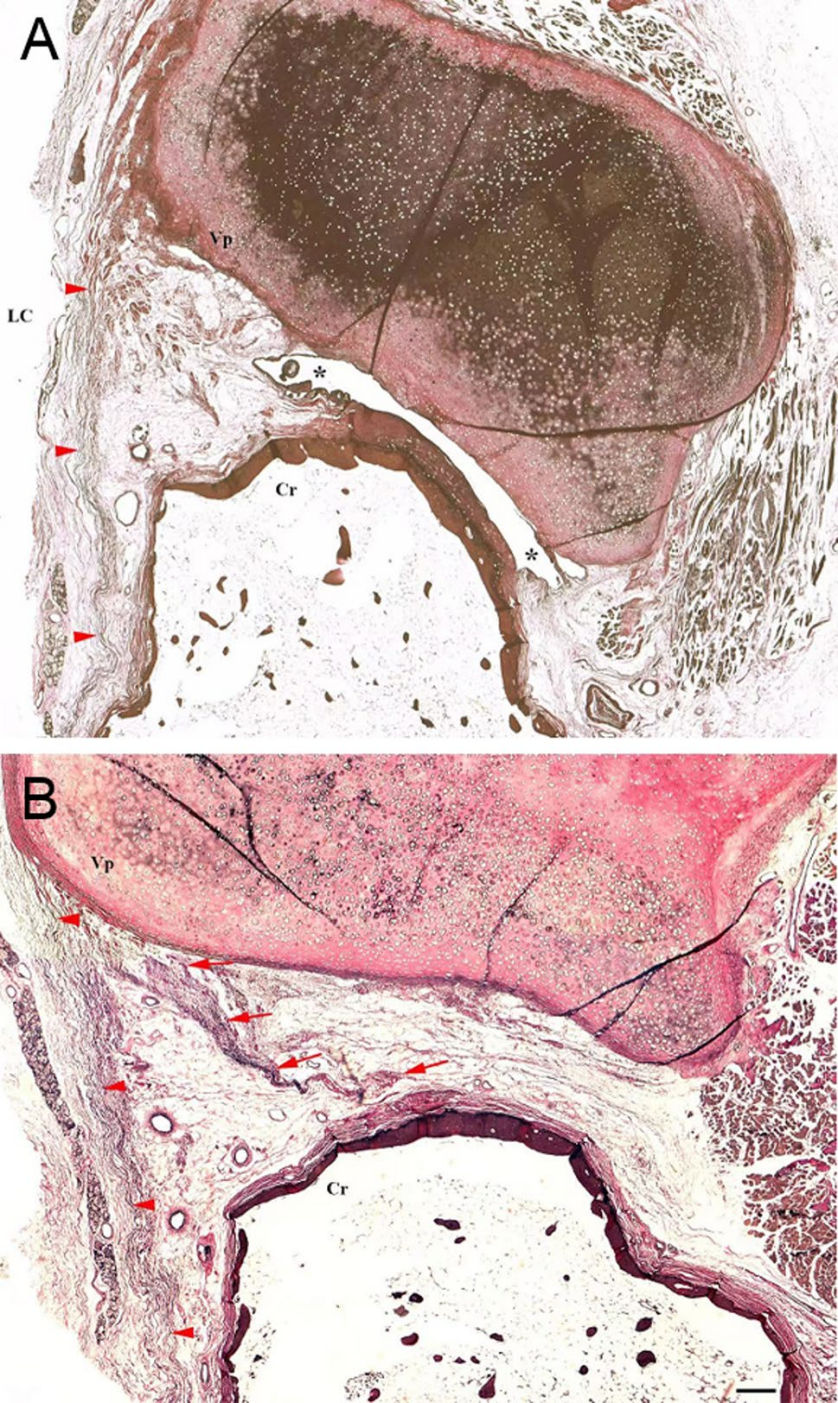
Two coronal sections stained with Verhoeff van Gieson method. **A** At the level of the anterior part of the cricoarytenoid joint (asterisks), the anterior cricoarytenoid ligament (arrowheads) extends between the base of the vocal process (Vp) of the arytenoid cartilage and the cricoid cartilage. LC indicates the side of the laryngeal cavity. **B** At the level anterior to the joint, the anterior cricoarytenoid ligament attaches to either anterior surface (arrowheads) or superior rim (arrows) of the cricoid cartilage (Cr). Bars = 1 mm

Two groups of the elastic fibers were located anterior–medially to the CAJ. One group was underneath the laryngeal mucosa and traversed along the medial (Fig. 1C), and attached onto the anterior–medial surface of the cricoid cartilage (Figs. 1C, 2A). Another group extended between the base of the vocal process and anterior–lateral surface and superior rim of the cricoid (Figs. 2B, 3), which was termed as the A-CAL in this study. A substantial space existed between the A-CAL and the CAJ cavity and mainly occupied by the adipose tissue (Figs. 2B, 3A). Thus, the A-CAL was an extra-capsule ligament. The elastic fibers were dominant in the part of the A-CAL close to the cricoid cartilage (Figs. 2B, 3) and appeared as loose and winding structures (Figs. 1E, 2B, 3B). The A-CAL was firmly attached onto the base of the vocal process of the arytenoid cartilage (Figs. 2B, 3).

### The elastic fibers posterior to the CAJ

In contrast to the anterior part of the CAJ, where the capsule was thin, loose and of few elastic fibers, the fibrous layer of the posterior capsule of the CAJ was strong and contained rich elastin fibers (Figs. 1C, 2C), which was named as the P-CAL in this study. Similar to the A-CAL, the elastic fibers were dominant in the part of the P-CAL close to the cricoid cartilage (Figs. 1C, 2C) but appeared taut or rigid (Fig. 1D). The P-CAL shared a common attachment with the posterior cricoarytenoid muscle onto the muscular process of the arytenoid cartilage (Fig. 2C).

## Discussion

In the present study, we classified the CAL into two parts: an extra-capsular A-CAL and an intra-capsular P-CAL. The both A-CAL and P-CAL contained rich elastic fibers. The elastic fibers of the A-CAL were orientated in both anterior–posterior and superior–inferior directions and under a relaxation status, whereas the elastic fibers of the P-CAL were arranged in a lateral–medial direction and under a taut status. The various fibrous configuration and status of the elastic fibers in the A-CAL and P-CAL may reflect different local tissue mechanical properties [10]. The results of this study re-confirm that the primary passive stabilizer of the CAJ is the P-CAL rather than the A-CAL.

The position and motions of the CAJ are controlled by both active (e.g., neuromuscular structures) and passive (noncontractile; e.g., ligaments, joint fibrous capsule) elements. The most intrinsic laryngeal muscles are attached to the muscular process of the arytenoid, whereas the passive elements are attached to both muscular and vocal processes. At rest, the intrinsic laryngeal muscles show a postural electrical activity, which maintains the position of the arytenoid cartilage and stabilizes the CAJ [5, 8]. It has been suggested that in addition to the rest tone of the intrinsic laryngeal muscles, a biomechanical balance between the anterior vocal ligament and the P-CAL may also contribute to the position of the arytenoid cartilage at quiet inspiration [6, 12, 13]. However, it remains unclear what role the A-CAL may play at the rest position and during motions of the CAJ. The findings of this study clearly demonstrated that the A-CAL was located anterior–medial–superiorly to the CAJ capsule, tortuously extended between the base of the arytenoid vocal process to the superior rim and anterior surface of the cricoid lamina, and orientated in a superiolateral-to-inferiomedial direction. Since the cadaveric vocal fold represents a position that the vocal fold adopts in a totally denervated larynx [12], the tortuous A-CAL indicated that the ligament was in a relaxed status and suggested that the A-CAL may not be involved in maintaining the cadaveric position of the CAJ. Its rich elastin components and specific fibrous orientation suggest that the A-CAL may play a role to limit an over-superiolateral movement of the arytenoid cartilage at the CAJ abduction. In addition, at phonation, the active force moves the arytenoid cartilage inferior–anterior–medially and confronts various passive forces associated with the vocal and muscular processes of the arytenoid cartilage, including the A-CAL, particularly those fibers orientated in an anterior–posterior direction [7].

It has been well-documented that the P-CAL is the primary passive stabilizer of the CAJ [2, 6]. This study further confirmed that the P-CAL was the strengthened posterior fibrous capsule of the CAJ and shared a common arytenoid attachment with the posterior cricoarytenoid muscle. The P-CAL contained both elastic and collagen fibers. The elastic fibers were dominant medially, whereas its collagen fibers were dominant laterally and intermingled with the common tendon of the posterior cricoarytenoid muscle. Such elastin–collagen composites and configuration suggest that the P-CAL is capable of greater extensions and contributes significantly to the biomechanical properties of the ligament at high stresses and strains [14]. For example, the studies on both cadavers and living subjects have demonstrated that the range of the vertical movement of the vocal process of the arytenoid cartilage at phonation is wider than that of the muscular process, which results in a visor-like downward vertical motion of the arytenoid cartilage at phonation [6, 7]. The P-CAL is the key posterior–lateral passive force to limit the mobility of the muscular process and stabilize the CAJ [6, 7].

This study has a limitation that the specimens were collected from the elderly cadavers. Although the elastin is a stable and persistence protein in human body, it has been reported that both elastin and collagen fibers in the larynx undergo degeneration and degradation during aging [15]. Thus, there may be an age difference of the ratio of the collagen to elastin and their configuration in the CAL. However, the aging effect on the elastin components of the CAJ may be quantitative rather than qualitative as no age-related changes were visible in the elastic attachments on the arytenoid cartilage as viewed by means of light microscopy [16].

## Conclusion

This study defined the fine configuration of the CAL, particularly its elastic fibers, which may help us to better understand the biomechanics of the CAJ motions, and differential diagnosis of CAJ disorders. The results of the study re-confirm that the P-CAL is the key posterior–lateral passive force to limit the mobility of the muscular process of the arytenoid cartilage and stabilize the CAJ, whereas the A-CAL may protect the CAJ from an over superior–lateral–posterior motion.

## Data Availability

The data that support the findings of this study are openly available in Figures 1–3 at 10.1007/s00405-023-08003-y.
